# Dietary Quality Evidenced by the Healthy Eating Index and Cardiovascular Disease Risk Factors in Kuwaiti Schoolchildren

**DOI:** 10.3390/nu16081243

**Published:** 2024-04-22

**Authors:** Abdulaziz Kh. Al-Farhan, Lorraine J. Weatherspoon, Karin A. Pfeiffer, Wei Li, Joseph J. Carlson

**Affiliations:** 1 Department of Food Science and Human Nutrition, Michigan State University, East Lansing, MI 48824, USA; weathe43@msu.edu (L.J.W.); carls122@msu.edu (J.J.C.); 2 The Public Authority for Applied Education and Training, The College of Nursing, Shuwaikh 23167, Kuwait; 3 Department of Kinesiology, Michigan State University, East Lansing, MI 48824, USA; kap@msu.edu; 4 Department of Dietetics & Nutrition, University of Arkansas for Medical Sciences, Little Rock, AR 72205, USA; wli@uams.edu; 5 Department of Radiology, Michigan State University, East Lansing, MI 48824, USA

**Keywords:** diet quality, CVD risk factors, schoolchildren

## Abstract

Background: Poor dietary quality is associated with adiposity and other risks of cardiovascular disease (CVD) in children. In Kuwait, although children’s food choices are a concern, no studies have evaluated dietary quality relative to the risk of CVD in Kuwaiti schoolchildren. This study hypothesized that dietary quality using the Healthy Eating Index (HEI) is associated with CVD risk factors in children and that there are associated sex differences. Objective: Our main objective was to evaluate the dietary quality of schoolchildren and investigate whether poor HEI scores are associated with CVD risk and if there are sex differences. Methods: This was a cross-sectional study of Kuwaiti fifth graders (*n* = 313; 53% girls; mean age = 10.4 ± 0.4 years) who completed an adapted Block Kids 2004 food frequency questionnaire. Anthropometric, blood pressure, and biochemical data were also measured. HEI-2010 and HEI-2015 scores were calculated. Statistics: A general linear model and logistic regression were applied, controlling for moderate–vigorous physical activity (MVPA) and screen time (ST). Results: The total HEI-2010 and HEI-2015 scores were 58 and 52 points, respectively; a trend analysis indicated that more girls than boys had poor (≤50 points) HEI-2015 scores (*p* < 0.063). The maximum scores for total vegetables (*p* < 0.001), dairy (*p* < 0.034), and fatty acids (*p* < 0.01) were significantly higher in girls, while the maximum scores for whole grains (*p* < 0.046) and protein (*p* < 0.006), but not sodium (*p* < 0.009), were higher in boys. Obesity was inversely associated with poor total HEI 2010 and HEI 2015 scores (OR: 0.347, 95% CI: 0.234 - 0.516, *p* < 0.001 and OR: 0.561, 95% CI: 0.391–0.805, *p* < 0.002, respectively). However, the correlation was lost after adjustment for possible confounding factors. Conclusions: Dietary quality for children overall in this study was low, and there was only a weak association between poor scores and elevated blood pressure and none between scores and obesity. These findings have public health implications and warrant further investigation and attention.

## 1. Introduction

The dietary quality of children affects their health and well-being. In particular, the lack of adherence to dietary guidelines increases the risk of obesity and other cardiovascular diseases (CVDs) in children [1]. In addition, physical inactivity and screen time are closely linked to the risk of adiposity and poor nutritional behavior in children [2]. It is also well known that an excessive intake of sodium, saturated fat, meat, and fast food, as well as a low intake of essential fatty acids, fruits, and vegetables, are inversely associated with CVD risk [3]. Cohort studies investigating children and adolescents at risk for CVD described their adverse blood lipid levels as being the same as those in adults and predicted risk for hypertension in adulthood for some children [4]. Economic transitions occurred during the early 1980s, in Kuwait [5], causing a shift in the nutritional behaviors and health of children from being undernourished and having an obesity prevalence of <1% in 1980 to having an obesity prevalence of 31% in 2012. This was associated with high caloric intakes, insufficient physical activity, and increased risk of prehypertension and adverse blood lipids among schoolchildren, particularly girls [6,7]. A recent study investigating the dietary patterns of Kuwaiti adults showed that high intakes of fast food and refined grains were associated with adiposity and hypertension [8]. However, it is not known if the same is true for Kuwaiti children. National nutritional guidelines, or specific dietary indices for evaluating dietary quality and behavior in children and adolescents, are also not available for the Kuwaiti population.

The Healthy Eating Index (HEI) is a dietary index designed to evaluate dietary quality. The HEI uses a density-based index (e.g., amount per 1000 kcal) to monitor dietary adherence to the Dietary Guidelines for Americans (DGA) [9]. Several HEI versions exist, including 2005, 2010, and 2015, which correspond with the 2015–2020 DGA edition. Unlike the HEI 2010 version, which combines calories from solid fats, alcoholic beverages, and added sugars as empty calories (these are known to contribute to adiposity), the HEI 2015 version separates saturated fat and added sugars that are suspected to increase the risk of atherosclerosis in children [10]. The HEI is a reliable and valid instrument [11] that can be applied for investigating cross-sectional associations between dietary quality and the risk of CVD [12,13]. A meta-analysis of 15 cohort studies (1,020,642 subjects) examined associations between dietary quality assessed using several dietary indices and the risks of all-cause mortality indicated that high HEI scoring was associated with significant risk reduction in CVD incidence and mortality (RR 0.78, 95% CI 0.75 to 0.81; *p* < 0.00001) by 22% [11]. Studies that have used the HEI to measure dietary quality in children have revealed that many children do not meet most of the guidelines, as indicated by the poor HEI scores. For example, a Canadian study among 4966 fifth graders reported a total HEI-2005 score of 62 points, indicating low vegetable, fruit, and grain intake [14]. Another study in the U.S. showed that metabolically healthy but obese adolescents who consumed more milk but fewer calories and less solid fats had good HEI-2005 scores and, consequently, a lower risk of CVD [15]. Research using data from the 2011–2012 National Health and Nutrition Examination Survey (NHANES) (*n* = 2857) indicated that children aged from 2 to 17 years had a total HEI-2010 score of 55 points, which was considered insufficient for meeting the guidelines, as well as a high sodium intake [16]. With respect to evaluating HEI 2015 in children and adolescents, data from NHANES 2015–2016 and 2017–2018 indicated total scores of 52 and 54 points [17,18], respectively, which may indicate a lower adherence to the DGA compared to when the previous HEI 2010 version was used. Another cross-sectional study using NHANES 2011–2014 reported associations between poor HEI-2015 scores and predicted 10-year CVD risk and heart age in adults [19]. Among the Kurdish population, a sub-cohort study (*n* = 295) observed that higher HEI 2015 scores and adherence to plant-based dietary patterns were associated with a lower risk of hypertension [20]. 

In Kuwait, no studies that examine dietary quality using the HEI or the relationship between dietary quality and CVD risk factors in children have been reported. Therefore, this study aimed to evaluate the HEI scores of school-age boys and girls and the relationship between poor HEI scores and CVD risk factors in schoolchildren. We expected that there would be sex differences in poor HEI scores associated with CVD risk factors based on previous findings that Kuwaiti female schoolchildren were at a significantly higher risk of being overweight, prehypertensive, and dyslipidemic compared to boys [6].

## 2. Methods

### 2.1. Study Design and Participants

**Figure 1:**
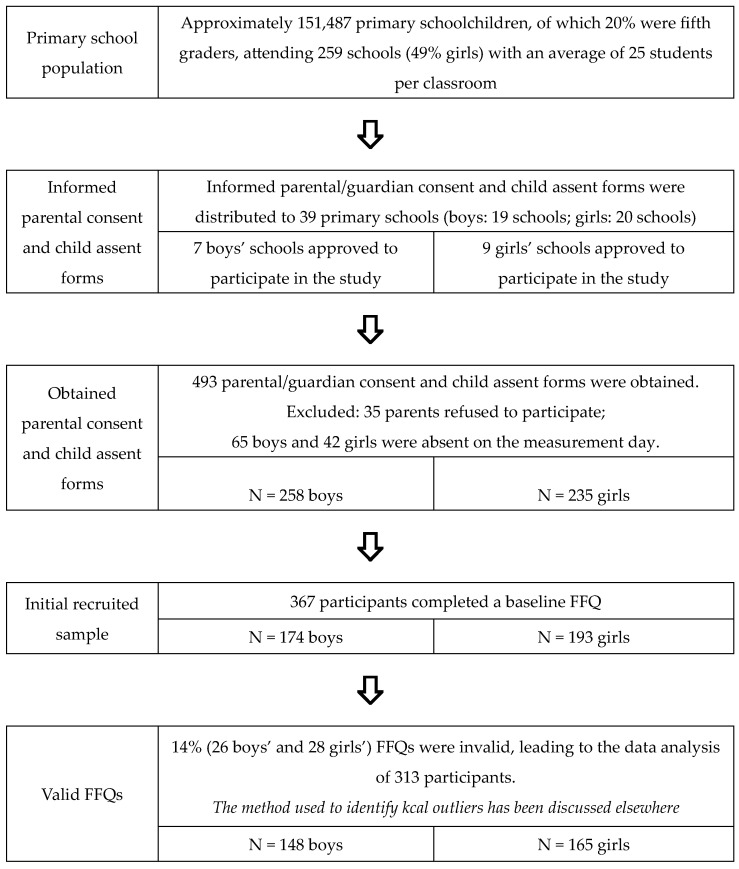
Flow figure of the study sample and recruitment and identifying kcals outliers [21,22].

This cross-sectional study recruited 313 fifth-grade schoolchildren (10.4 ± 0.4 y of age; 53% girls) in Kuwait. The study used an opportunistic cluster sampling technique design, involving two steps that included inviting primary schools within six governorates in Kuwait (Capital, Hawalli, Farwaniya, Mubarak Al-Kabeer, Ahmadi, and Jahra) to participate in the study. Next, participants provided parental consent and child assent for the study, and children who opted not to perform the tests or were absent on the day of measurement were excluded (Figure 1). The study was approved by the Institutional Review Board of Michigan State University (MSU) (IRB # 17-893), Ministry of Health Research Ethics Committee (REC # 561), and the Department of Educational Research (DER # 23641) in Kuwait.

### 2.2. Measurements

The dietary data collection procedure was part of a comprehensive study that assessed cardiovascular disease risk factors in Kuwaiti fifth graders between February and March 2018. The study participants underwent anthropometric, blood pressure, and biochemical assessments at the school site, as detailed elsewhere [6]. Standing height and body weight were measured, and body mass index (BMI) was calculated and then converted to BMI Z-scores using the World Health Organization’s (WHO) (2007) LMS cutoff point percentile reference [23], as well as ≥+1 SD for overweight and ≥+2 SD for obesity [24]. Percentage body fat was estimated via bioelectrical impedance (Tanita BC-534) and waist circumference (WC) was measured to the nearest 0.1 cm using a Gulick measuring tape. Blood samples were obtained from participants in a non-fasted state through a finger prick (40 µL) using heparinized capillary tubes. Blood samples were analyzed using a valid portable analyzer, CardioCheck Plus (version 1.09; Maria Stein, OH, USA), which was calibrated at each school site prior to measurement. Cardiocheck Pluse analyzed TC, HDL-C, and triglycerides. It is employed to calculate LDL-C ((LDL = TC − HDL + TG/5), the TC:HDL ratio, and non-HDL over a period of 90 s, and has been used in Kuwaiti [6] and US schoolchildren [25]. Resting systolic (SBP) and diastolic (DBP) blood pressure were manually assessed using a stethoscope and a standard BP aneroid, with an appropriately sized inflatable cuff on the subject’s right arm. Children at risk of elevated blood pressure were identified based on the 2017 Clinical Practice Guidelines for Screening and Management of High Blood Pressure in Children and Adolescents aged 1 to <13 years. Cut off points include Elevated BP (≥90th percentile to <95th percentile or 120/80 mm Hg to <95th percentile, whichever is lower), Stage 1 HTN (≥95th percentile to <95th percentile + 12 mmHg, or 130/80 to 139/89 mm Hg, whichever is lower), and Stage 2 HTN (≥95th percentile + 12 mm Hg or ≥140/90 mm Hg, whichever is lower) [26], as illustrated in Table 1. Dietary intake was assessed using a reliable and culturally modified version of the Block Kids 2004 food frequency questionnaire (FFQ), translated into Arabic and developed by the researcher and NutritionQuest for use with Kuwaiti children [21]. The adapted version consists of eight pages that evaluate the frequency and quantity of children’s consumption of 72 food and beverages items during the past week. The data dictionary and nutrient analyses were adjusted after inclusion, or some foods and beverage items were emphasized to maintain the validity of the nutrient data. The self-reported FFQ was administered to the study participants, who were guided by trained staff with example pictures of typical portion sizes and food models used to estimate portion size. The FFQs were analyzed, and raw nutrient and food group data were generated using Block Dietary Data Systems (Berkeley, CA, USA). The raw nutrients were quantified and transformed into daily intakes of energy and nutrients in standard measurement units. Further details regarding the adapted FFQ development and testing are discussed elsewhere [21]. The nutritional data were derived from food groups, macronutrients, and micronutrients according to the DGA 2015–2020, including the dietary reference intake for individuals by age and gender [27]. Physical activity (PA) and screen time (ST) behaviors were self-reported by participants using the Youth Risk Behavior Survey questions [28]. The physical activity questionnaire (translated into Arabic) assesses the number of days that a child engaged in 60 min of moderate-to-vigorous PA (MVPA) during the past week. The ST questionnaire estimates the number of hours that children spend during week days and weekends spent in front of the televisions, with video games, and on computers according to specific formula [2]. The surveys were administered by trained nursing and nutrition students from the College of Nursing and Health Sciences of the Public Authority for Applied Education and Training in Kuwait, following established procedures of a school-based program for promoting physical activity, nutrition and cardiovascular health in schoolchildren [29].

### 2.3. Dietary Indices

In this study, both the HEI-2010 and the HEI-2015 scoring systems were applied and analyzed to account for the updated moderation components in HEI-2015 and to understand participants’ dietary status in both HEI versions.

The HEI-2010 has a total of 12 components, 9 of which reflect the adequacy of fruit, vegetables, whole grains, dairy, and protein (including seafood and plant-based proteins as well as polyunsaturated and monounsaturated fatty acids); the other 3 refer to moderation (refine carbohydrates, sodium and “empty calories” [9].

The 2015 update of the HEI by the National Cancer Center (i.e., HEI-2015) to 13 components included adding saturated fat and added sugar as separate components to align with the latest DGA 2015–2020 recommendations [27]. All other components remained the same as the HEI-2010.

In the standards score calculations, the HEI-2010 and HEI-2015 effectively use the amount per 1000 calories or percent of total calories as well as gender and age comparisons. A high score in the adequacy components shows alignment with national recommendations, while a minimum score above zero shows that national recommendations have not been met for the moderation score. A HEI “maximum” is allocated when adequacy intake levels are standard or higher and when intake levels are standard or lower for moderation components. The mean daily consumption frequency from HEI components ranged from 0 to 10) with a total overall score of 0 to 100 [30,31,32]. If the HEI is over 80, it is categorized as “good”, and if the score is 50 or lower, it is considered “poor” [33].

SPSS version 24 was used to generate the total HEI-2010 scores from FFQ data but calculating HEI 2015 scores required adding the “empty calories”, “saturated fat” and “added sugars” components. More details on these score calculations are available on the National Cancer Institute website [34].

### 2.4. Statistical Analysis

SPSS version 24 (IBM Corp., Armonk, NY, USA) was used for data analysis, and the results are illustrated as mean ± standard deviation (SD) or standard error (SE) at a *p* < 0.05 significance level. Power sampling size calculation (1-β, 0.95; α, 0.05, critical *t* = 1.97143) using G*Power software 3.1.9.3: Heinrich Heine Universitat Düsseldorf, 2017, indicated a minimum of 210 participants using a *t*-test for mean differences, in addition to goodness-of-fit χ^2^ tests for comparing two independent groups, which estimated a minimum of 220 participants. A Q–Q box plot was applied to test for the normality of variable distribution. Positively skewed triglycerides (TGs) were logarithmically transformed. Demographic characteristics, HEI standard scores per 1000 kcal, and maximum scores were analyzed using a *t*-test and chi-square test. We applied a general linear model with adjustments for total calories, moderate-to-vigorous physical activity (MVPA), and ST to compare mean absolute nutrient intake levels and mean CVD risk factors. Pearson’s correlation evaluated the relationship between HEI components and CVD risk factors. Binary logistic regression was applied to investigate the likelihood of associations between poor HEI scores and CVD risk factors, controlling for sex, MVPA, and ST.

## 3. Results

Table 1 presents the demographic, anthropometric, and biometric characteristics of participants. No statistical differences were found in the prevalence of overweight (22.8%) and obesity (41%) between sexes. Girls had significantly higher mean DBP, TGs, and elevated BP compared to boys. A significant trend showed that more boys were meeting the MVPA recommendations, while girls significantly met the ST recommendations. 

Table 2 compares the mean daily dietary intakes by sex. Boys had a significantly higher mean calorie intake than girls. However, adjusting for total kcals revealed fewer statistical differences in mean daily intake levels between sexes. 

Table 3 summarizes the results of evaluating total and component scores of HEI-2010 and gender differences. Participants’ overall mean total HEI-2010 score was 58 points, and around 14% of the study participants’ total HEI-2010 scores were considered poor (≤50 points), especially for girls, but this number was not statistically significant. With regard to adequacy components, total vegetables, dairy, and fatty acid maximum scores were statistically higher in girls, while the maximum scores for whole grain and protein components were statistically higher in boys. For moderation components, boys’ sodium maximum score was significantly lower than that of girls.

The HEI-2015 results presented in Table 4 reveal an overall mean total score of 52 points. Approximately 36% of participants’ total scores were below 50 points. A trend analysis showed that around 41% of girls scored 50 points or lower compared to boys (31.1%) (*p* = 0.063). There were no statistical differences for gender detected in the total HEI-2015 score or in the maximum scores for the moderation components of added sugars and saturated fat between sexes.

Table 5 shows correlations between the total and mean HEI-2010 and HEI-2015 component scores and mean CVD risk factors of the study participants. Mean SBP was positively correlated with the maximum score for the fatty acid component and was inversely correlated with the maximum score for the sodium component. Additionally, elevated BP was positively weakly correlated with poor HEI 2010 and 2015 total scores (*r* = 0.121; *p* = 0.035; *r* = 0.146; *p* = 0.011, respectively). MVPA was positively correlated with the maximum score of the total fruit component and total HEI-2010 scores and was inversely correlated with the maximum score for the saturated fat component. An inverse correlation between ST and maximum score for the empty calories component was detected. However, after the adjustments for sex, correlations between HEI and mean CVD risks were no longer significant. 

With regard to the association between the poor total HEI scores and CVD risk factors shown in Table 6, a binary logistic regression, adjusted for sex, MVPA, and ST, indicated an adverse association between the unadjusted obesity and HEI 2010 and 2015 poor total scores, which was attenuated after controlling for sex, MVPA, and ST. In addition, there were trends of associations between unadjusted elevated BP and poor HEI 2010. Total scores diminished after controlling for covariates, however, a weak association was observed between poor HEI 2015 total score and elevated BP after controlling for covariates.

## 4. Discussion

Limited studies have reported on the dietary quality of Kuwaiti schoolchildren and its relationship with CVD risk factors. Our study findings revealed that poor HEI 2015 score is weakly associated with elevated blood pressure, influenced by sex. Around 40% of participants had poor mean total HEI-2010 and HEI-2015 scores lower than 50 points, and no participants achieved a good HEI score of 80 points or higher. As we expected, a poor dietary quality associated with risks of CVD was more pronounced in girls than boys. A similar finding among girls of the same age group in Spain was reported in a previous study [37]. 

Our estimated mean HEI-2010 score of 58 points was lower than that reported in similarly aged schoolchildren. A 2012 study involving fifth graders (*n* = 210) in Michigan, USA, reported a total HEI-2010 score of 62 points, where only 2.5% had good HEI-2010 scores [30]. On the other hand, a study among 2–17-year-old children and adolescents (*n* = 2857) from NHANES 2011–2012 reported a slightly lower total HEI-2010 score (55 points) [16].

We compared our findings for the total HEI-2015 with the available studies, considering variations in age categories, sample size, and race and ethnicity. We observed similar mean total HEI scores with those from the NHANES data report (2015–2016, 2 to 18 years of age), which reported a total HEI score of 52 points [25]. However, based on NHANES data (2009–2010, 2011–2012, and 2013–2014), Thomson et al. described the dietary quality of US children (2 to 18 years, *n* = 9000) and reported a mean total HEI 2015 score of 54.9 [38], higher than the score obtained in our findings. Salas-González et al. investigated dietary quality in 854 Spanish schoolchildren (3 to 18 years of age) and reported a mean total HEI-2015 score of 59.3, also higher than our findings. Nevertheless, among the specific age groups for children (2–5, 6–11, and 12–18) and BMI categories, the NHANES data cycle from 2009 to 2014 showed lower total HEI scores among children aged 12 to 18—46.4 points (underweight), 52 points (normal weight), 51.5 points (overweight), and 52.2 points (obese) compared to other age groups. The mean total HEI scores for children in the obese category were especially relevant to our estimated mean total HEI 2015 score. However, in children aged 6 to 11 years old, the mean total HEI scores, with the exception of those for children in the underweight category, were better than those obtained in our findings [39]. More recent NHANES data (2017–2018) on children aged 9 to 13 years show a mean total HEI 2015 score of 53, higher than the score obtained in our findings, in addition to better component scores for whole fruit, dairy, fatty acids, refined grains, and sodium [18]. This may imply that poor dietary quality among children in Kuwait is related to excessive body weight; it should be noted that 42% of our study participants were obese. Moreover, the HEI can be a useful dietary tool to determine dietary quality status and demonstrate contrasts with other races and ethnicities in children. 

Cohort studies in children and adolescents observed increased levels of SBP and TG among girls than in boys, starting from the age of 11 years [40]. Consistent with our study’s findings that poor HEI scores were associated with elevated BP risk, particularly in girls, Salas-González et al. also found that poor HEI scores were associated with higher insulin resistance in girls than in boys [37]. Nevertheless, in 2018, Hooshmand F. et al. used baseline data to assess the relationship between the modified Healthy Eating Index (mHEI) and the development of metabolic syndrome (MetS) (elevated fasting blood glucose and TGs and low HDL) among healthy Iranian children and adolescents aged 6 to 18 years (*n* = 424, 57% girls). The mean total HEI score for the study participants was 55.9 and was not related to MetS, nor were sex differences detected when considering the mean BP [41]. 

We did not detect correlations between mean blood lipid and HEI component scores. This may be related to sample size and the lipid levels of our sample as well as the fluctuating blood lipid levels in children and adolescents related to age and sex [40]. Hooshmand’s cohort data for Iranian children and adolescents indicated that 11% of participants, mostly males, developed MetS, but this was not associated with HEI scores [41].

Research shows that sedentary lifestyle behaviors affect children’s dietary patterns [2] and related CVD risks [42]. Therefore, the DGA combines PA recommendations to promote optimal nutrition and disease prevention [27]. Drenowatz et al. investigated the combined association of PA or ST with HEI 2010 on risks of CVD among 210 U.S. fifth graders. The study observed an association between high HEI scores (>61 points) and high PA category; however, this did not directly affect the risk of CVD [30]. In our sample, the estimated total HEI 2010 score of 62 points was correlated with MVPA, which may explain the fact that physical activity influences children’s dietary quality. Among 214 U.S. schoolchildren (3rd to 5th graders), Montoye et al. observed that only 24% of children met screen time recommendations, which was significantly correlated with their junk food intake [2]. Similar to our findings, meeting ST recommendations was low (17%) and was correlated with a poor empty calories score. 

Recent nutritional and public health guidelines and policies include the DGA [17] and the WHO [43], which focus on promoting healthy lifestyle behavior in children to prevent childhood obesity and nutrition-related diseases on a global scale. There is evidence that school-based physical and education programs are effectively intervening through establishing guidelines and improving children’s dietary and activity behaviors all around the world [44]. For example, an experimental study in the US compared a school- and web-based intervention program consisting of eight nutrition and PA lessons among 181 fifth graders. The first group received the lesson during PE classes and through a newsletter sent to parents; the second group received similar lessons provided by mentors and school environment components. The study also measured participants pre and post dietary, PA, and CVD risks, and found significant improvements in dietary and nutrition PA among the second group [45]. Such programs are needed and will hence also be beneficial for schoolchildren in Kuwait. 

A main strength of the current study was utilizing the HEI scoring system to evaluate the dietary quality and related risk of CVD by considering potential influences of MVPA and screen time and applying it to a sample of Kuwaiti schoolchildren, previously a gap in the literature. In addition, this study also used a reliable tailored FFQ for use in Kuwaiti children. Our study used the opportunistic cluster sampling technique; nevertheless, the study was not interventional, did not include a follow-up, and was not generalizable or representative of all Kuwaiti fifth graders. The sample size for blood lipids was lower than that for other variables due to insufficient blood volume, technical issues with the analyzer, or because some children opted out of the blood lipid test. It should be noted that dietary misreporting in children is influenced by their cognitive ability to remember what they have eaten and to estimate portion sizes, regardless of sex [46]. In addition, dietary indices in general may not effectively predict chronic diseases related to methodology, combining measures, or using single components that may affect the analysis, also related to complexity of the diet [47]. Moreover, biological factors such as pubertal status were not accounted for in this study.

## 5. Conclusions

This was the first study to report the HEI scores of Kuwaiti school boys and girls and their associated risk of CVD. Most children’s HEI-2010 and HEI-2015 scores were poor, and there was only a weak association between poor scores and elevated blood pressure and none between scores and obesity. The findings of this study suggest the need for intervention programs to improve Kuwaiti children’s dietary behavior and the potential risk of CVD.

## Figures and Tables

**Table 1:** Demographic characteristics and anthropometrics, blood pressure, blood lipids, and covariates (moderate-to-vigorous physical activity (MVPA) and screen time (ST)) of fifth-grade Kuwaiti boys and girls.

Characteristic	Overall (*n* = 313)	Boys (*n* = 148)	Girls (*n* = 165)	*p*-Value
City				
Capital	11.8%	9.5%	13.9%	0.22
Hawalli	38.7%	37.8%	39.4%	0.77
Farwaniya	5.8%	6.8%	4.8%	0.47
Mubarak Al-Kabir	22.7%	25.7%	20.0%	0.23
Ahmadi	10.5%	3.4%	17.0%	0.001
Jahra	10.5%	16.9%	4.8%	0.001
Age (years)	10.45 ± 0.38	10.44 ± 0.35	10.46 ± 0.40	0.572
Height (cm)	142.06 ± 6.62	141.3± 7.07	142.4 ± 6.12	0.104
Weight (kg)	44.48 ± 13.11	43.93 ± 14.22	44.98 ± 12.05	0.479
BMI (kg/m^2^)	21.74 ± 4.99	21.59 ± 5.32	21.87 ± 4.68	0.631 *
BMI Z-score	1.42 ± 0.07	1.47 ± 0.11	1.39 ± 0.10	0.103 *
+1 Z-score	22.8%	18.2%	26.8%	0.071
+2 Z-score	42%	43.9%	40.2%	0.511
WC (cm)	69.9 ± 0.78	69.5 ± 1.21	70.3 ± 1.0	0.466 *
BF%	28.5 ± 0.57	27.4 ± 0.95	29.5 ± 0.67	0.468 *
SBP mmHg	106.3 ± 0.69	103.9 ± 0.99	108.3 ± 0.94	0.058 *
DBP mmHg	68.01 ± 0.55	65.1 ± 0.73	70.5 ± 0.76	0.001 *
Elevated BP				
≥90th to <95th percentiles or 120/<80 to 129/<80 mm Hg	16% (*n* = *49*)	5.2% (*n* = *16*)	10.7% (*n* = *33*)	0.029
Stage 1 HTN				
95th percentiles or 130/80 to 139/89 mmHg Stage 2 HTN	2.9% (*n* = *9*)	1.3% (*n* = *4*)	1.6% (*n* = *5*)	0.881
95th + 12 mmHg or ≥140/90 mm Hg	0.7% (*n* = *2*)	0.3% (*n* = *1*)	0.3% (*n* = *1*)	0.930
TC (mg/dL)	152.9 ± 2.2	149.5 ± 2.7	156 ± 3.4	0.837 *
LDL (mg/dL)	82.6 ± 1.81	82.9 ± 2.66	82.4 ± 2.47	0.505 *
HDL (mg/dL)	51.5 ± 0.94	51.6 ± 1.18	51.4 ± 1.45	0.383 *
None-HDL (mg/dL)	100.7 ± 1.7	99.2 ± 2.4	102.1 ± 2.5	0.936 *
TC:HDL	3.0 ± 0.05	2.9 ± 0.07	3.1 ± 0.08	0.399 *
Triglycerides (mg/dL)	98.4 ± 3.2	90.1 ± 4.1	105.7 ± 4.9	0.029 *
Covariate				
MVPA (d/wk)	2.89 ± 2.34	3.37 ± 2.36	2.47 ± 2.24	0.001
% met MVPA	19.3%	24%	15.2%	0.054
ST (h/day)	4.72 ± 2.68	4.97 ± 2.56	4.50 ± 2.77	0.119
% met ST ≤ 2 h/d	17.4%	9.6%	24.2%	0.001

Descriptive statistics—general linear model adjusted * for MVPA and ST presented as mean (SD) or SE, *p* < 0.05 for differences between sexes: BMI, body mass index; BF%, percentage body fat; WC, waist circumference; SBP, systolic blood pressure; DBP, diastolic blood pressure; BP, blood pressure; HTN, hypertension; TC, total cholesterol; LDL, low-density lipoprotein; HDL, high-density lipoprotein; MVPA, moderate-to-vigorous physical activity (number of days over past week (7 days) meeting > 60 min); ST, screen time (h/day). Italics indicates controlled variables.

**Table 2:** Mean food group, macronutrient, and micronutrient daily intake levels of fifth-grade Kuwaiti boys versus girls.

Nutrition Variable	Overall (*n* = 313)	Boys (*n* = 148)	Girls (*n* = 165)	*p*-Value	*p*-Value *
Food Group					
Fruits (cup)	2.36 ± 0.080	2.56 ± 0.12	2.19 ± 0.10	0.020	0.487
Vegetables (cup)	1.81 ± 0.05	1.81 ± 0.08	1.80 ± 0.07	0.963	0.011
Dairy (cup)	1.67 ± 0.04	1.70 ± 0.06	1.65 ± 0.06	0.311	0.027
Whole Grains (ounces)	0.63 ± 0.02	0.68 ± 0.03	0.59 ± 0.02	0.047	0.785
Macronutrient (g)					
Total Calories	2460.29 ± 52.2	2652.59 ± 76.9	2287.38 ± 68.5	0.001	
Total Fat	92.08 ± 2.25	99.82 ± 3.34	85.14 ± 2.96	0.001	0.892
Saturated Fat	29.21 ± 0.66	31.3 ± 0.95	27.3 ± 0.91	0.002	0.306
*Trans*-fat	8.15 ± 0.23	8.82 ± 0.33	7.55 ± 0.33	0.008	0.653
*n*-6 FAs	19.12 ± 0.62	21 ± 0.97	17.4 ± 0.76	0.004	0.829
*n*-3 FAs	1.67 ± 0.041	1.82 ± 0.062	1.53 ± 0.053	0.001	0.377
Cholesterol (mg)	305.1 ± 8.22	338.3 ± 12.07	275.3 ± 10.73	0.001	0.029
Carbohydrates	335.7 ± 7.10	359.1 ± 10.64	314.6 ± 9.25	0.002	0.414
Total Sugar	162.6 ± 4.04	175.6 ± 6.38	150.9 ± 4.94	0.002	0.790
Added Sugar (tsp) ^“^	22.1 ± 0.68	24 ± 1.09	20.5 ± 0.84	0.012	0.373
Total Fiber	22.08 ± 0.55	23.6 ± 0.82	20.7 ± 0.72	0.010	0.449
Fiber per 1000 kcals	8.9 ± 0.11	8.8 ± 2.00	8.9 ± 2.00	0.655	0.418
Protein	83.2 ± 1.85	91.2 ± 2.74	76.2 ± 2.38	0.001	0.028
Micronutrient					
Calcium (mg)	964.04 ± 22.08	1008.8 ± 30.34	923.8 ± 31.58	0.054	0.031
Magnesium (mg)	332.8 ± 8,02	357.9 ± 11.97	310.1 ± 10.51	0.003	0.527
Potassium (mg)	3010.8 ± 66.7	3201.3 ± 108.41	2840.0 ± 97.15	0.007	0.369
Sodium (mg)	3664.2 ± 79.5	3920.3 ± 113.6	3434.5 ± 108.4	0.002	0.695
Vitamin D (IU) ^^^	140.7 ± 4.29	145.8 ± 6.28	136.2 ± 5.87	0.267	0.556

General linear model (* *p*-Value controlled for total calories); data are presented as mean ± SE, *p* < 0.05 for differences between sexes: *n*-6 FAs, omega 6, linoleic acid; *n*-3 FAs, omega 3, linolenic acid. ^“^ One teaspoon equivalent = 4.2 g of sugar [35]. ^^^ IU (international unit), 1 IU of vitamin D = 0.025 µg.

**Table 3:** Healthy Eating Index-2010 (HEI-2010) (12 components, 0–100 points) of Kuwaiti fifth-grade boys (*n* = 148) vs. girls (*n* = 165) ^1^.

Component	Maximum Points	Standard for Maximum Score	Standard for Minimum Score of Zero	Participants’ Standard Scores per 1000 kcal			Participants’ Maximum Points		
Adequacy:				Boys	Girls	*p*-Value	Boys	Girls	*p*-Value
Total Fruit ^2^	5	≥0.8 cup equivalent per 1000 kcal	No fruit	0.99 ± 0.045	0.98 ± 0.043	0.911	3.33 ± 0.07	3.29 ± 0.07	0.655
Whole Fruit ^3^	5	≥0.4 cup equivalent per 1000 kcal	No whole fruit	0.54 ± 0.036	0.53 ± 0.031	0.921	3.09 ± 0.096	3.12 ± 0.090	0.841
Total Vegetables ^4^	5	≥1.1 cup equivalent per 1000 kcal	No vegetables	0.69 ± 0.028	0.78 ± 0.026	0.016	2.30 ± 0.069	2.61 ± 0.061	0.001
Greens and Beans ^4^	5	≥0.2 cup equivalent per 1000 kcal	No dark leafy vegetables or greens and beans	0.20 ± 0.016	0.23 ± 0.016	0.161	2.56 ± 0.161	2.64 ± 0.153	0.717
Whole Grains	10	≥1.5 oz equivalent per 1000 kcal	No whole grains	0.26 ± 0.011	0.26 ± 0.011	0.823	4.09 ± 0.170	3.63 ± 0.151	0.042
Dairy ^5^	10	≥1.3 cup equivalent per 1000 kcal	No dairy	0.66 ± 0.020	0.72 ± 0.024	0.036	4.56 ± 0.123	4.96 ± 0.136	0.034
Total Protein Foods ^6^	5	≥2.5 oz equivalent per 1000 kcal	No protein foods	2.44 ± 0.070	2.19 ± 0.067	0.011	3.23 ± 0.058	2.99 ± 0.065	0.006
Seafood and Plant Proteins ^7^	5	≥0.8 oz equivalent per 1000 kcal	No seafood or plant proteins	1.49 ± 0.083	1.33 ± 0.070	0.131	3.58 ± 0.059	3.49 ± 0.061	0.291
Fatty Acids ^8^	10	(PUFAs + MUFAs)/SFAs > 2.5	(PUFAs + MUFAs)/SFAs ≤ 1.2	0.81 ± 0.029	0.96 ± 0.037	0.001	1.26 ± 0.099	1.73 ± 0.149	0.010
Moderation:									
Refined Grains	10	≤1.8 oz equivalent per 1000 kcal	≥4.3 oz equivalents per 1000 kcal	2.80 ± 0.050	2.83 ± 0.050	0.620	7.27 ± 0.15	7.15 ± 0.16	0.578
Sodium	10	≤1.1 g per 1000 kcal	≥2.0 g per 1000 kcal	1.49 ± 0.018	1.50 ± 0.018	0.683	9.27 ± 0.202	8.36 ± 0.271	0.009
Empty Calories ^9^	20	≤19% of energy	≥50% of energy	28.56 ± 0.46	28.69 ± 0.44	0.845	13.98 ± 0.723	13.86 ± 0.254	0.734
					Total HEI Score		58.54 ± 7.02	57.86 ± 7.81	0.429
					Good Score ≥ 80		0%	0%	
					Poor Score ≤ 50		12.2%	15.8%	0.374

^1^ Independent *t*-test and chi-square test data presented as mean ± SE or SD (total HEI score), *p* < 0.05 for differences between sexes. ^2^ Includes fruit juice. ^3^ Includes all forms except juice. ^4^ Includes any beans and peas (called legumes in HEI-2005) not counted as total protein foods (called meat and beans in HEI-2005). ^5^ Includes all milk products, such as fluid milk, yogurt, and cheese, and fortified soy beverages. ^6^ Beans and peas are included here (and not with vegetables) when the total protein food (called meat and beans in HEI-2005) standard is otherwise not met. ^7^ Includes seafood, nuts, seeds, and soy products (other than beverages), as well as beans and peas counted as total protein foods. ^8^ Ratio of polyunsaturated fatty acids (PUFAs) and monounsaturated fatty acids (MUFAs) to saturated fatty acids (SFAs). ^9^ Calories from solid fats, alcohol, and added sugars; the threshold for counting alcohol is >13 g/1000 kcal [9].

**Table 4:** Healthy Eating Index-2015 (HEI-2015) (13 components, 0–100 points) of Kuwaiti fifth-grade boys (*n* = 148) vs. girls (*n* = 165) ^1^.

Component	Maximum Points	Standard for Maximum Score	Standard for Minimum Score of Zero	Participants’ Standard Scores per 1000 kcal			Participants’ Maximum Points		
Adequacy:				Boys	Girls	*p*-Value	Boys	Girls	*p*-Value
Total Fruit ^2^	5	≥0.8 cup equivalent per 1000 kcal	No fruit	0.99 ± 0.045	0.98 ± 0.043	0.911	3.33 ± 0.07	3.29 ± 0.07	0.655
Whole Fruit ^3^	5	≥0.4 cup equivalent per 1000 kcal	No whole fruit	0.54 ± 0.036	0.53 ± 0.031	0.921	3.09 ± 0.096	3.12 ± 0.090	0.841
Total Vegetables ^4^	5	≥1.1 cup equivalent per 1000 kcal	No vegetables	0.69 ± 0.028	0.78 ± 0.026	0.016	2.30 ± 0.069	2.61 ± 0.061	0.001
Greens and Beans ^4^	5	≥0.2 cup equivalent per 1000 kcal	No dark leafy vegetables or greens and beans	0.20 ± 0.016	0.23 ± 0.016	0.161	2.56 ± 0.161	2.64 ± 0.153	0.717
Whole Grains	10	≥1.5 oz equivalent per 1000 kcal	No whole grains	0.26 ± 0.011	0.26 ± 0.011	0.823	4.09 ± 0.170	3.63 ± 0.151	0.042
Dairy ^5^	10	≥1.3 cup equivalent per 1000 kcal	No dairy	0.66 ± 0.020	0.72 ± 0.024	0.036	4.56 ± 0.123	4.96 ± 0.136	0.034
Total Protein Foods ^6^	5	≥2.5 oz equivalent per 1000 kcal	No protein foods	2.44 ± 0.070	2.19 ± 0.067	0.011	3.23 ± 0.058	2.99 ± 0.065	0.006
Seafood and Plant Proteins ^7^	5	≥0.8 oz equivalent per 1000 kcal	No seafood or plant proteins	1.49 ± 0.083	1.33 ± 0.070	0.131	3.58 ± 0.059	3.49 ± 0.061	0.291
Fatty Acids ^8^	10	(PUFAs + MUFAs)/SFAs > 2.5	(PUFAs + MUFAs)/SFAs ≤ 1.2	0.81 ± 0.029	0.96 ± 0.037	0.001	1.26 ± 0.099	1.73 ± 0.149	0.010
Moderation:									
Refined Grains	10	≤1.8 oz equivalent per 1000 kcal	≥4.3 oz equivalent per 1000 kcal	2.80 ± 0.050	2.83 ± 0.050	0.620	7.27 ± 0.15	7.15 ± 0.16	0.578
Sodium	10	≤1.1 g per 1000 kcal	≥2.0 g per 1000 kcal	1.49 ± 0.018	1.50 ± 0.018	0.683	9.27 ± 0.202	8.36 ± 0.271	0.009
Added Sugars	10	≤6.5% of energy	≥26% of energy	14.98 ± 0.47	15.17 ± 0.43	0.768	4.43 ± 0.197	4.46 ± 0.189	0.901
Saturated Fats ^9^	10	≤8% of energy	≥16% of energy	10.63 ± 0.104	10.62 ± 0.113	0.954	3.47 ± 0.111	3.44 ± 0.120	0.850
					Total HEI Score		52.50 ± 5.68	51.92 ± 6.17	0.385
					Good Score ≥ 80		0%	0%	
					Poor Score ≤ 50		31.1%	41.2%	0.063

^1^ Independent *t*-test and chi-square test data presented as mean ± SE or SD (total HEI score), at *p* < 0.05 for differences between sexes. ^2^ Includes fruit juice. ^3^ Includes all forms except juice. ^4^ Includes any beans and peas (called legumes in HEI-2005) not counted as total protein foods (called meat and beans in HEI-2005). ^5^ Includes all milk products, such as fluid milk, yogurt, and cheese, and fortified soy beverages. ^6^ Beans and peas are included here (and not with vegetables) when the total protein food (called meat and beans in HEI-2005) standard is otherwise not met. ^7^ Includes seafood, nuts, seeds, soy products (other than beverages), and beans and peas counted as total protein foods. ^8^ Ratio of polyunsaturated fatty acids (PUFAs) and monounsaturated fatty acids (MUFAs) to saturated fatty acids (SFAs). ^9^ In 2005, the sodium and saturated fats components had three standards each, corresponding to scores of 0, 8, and 10 points [36].

**Table 5:** Pearson’s correlation between mean HEI-2010 and HEI-2015 component scores and mean CVD risk factors in schoolchildren.

	BMI	BMI-Z	WC	BF%	SBP	DBP	TC	HDL	LDL	TC:HDL	Non-HDL	TG	MVPA	ST
Adequacy Components														
Total Fruit	−0.054	−0.064	−0.042	−0.079	−0.063	−0.039	−0.004	0.038	−0.004	−0.021	−0.005	−0.074	0.229 **	−0.030
Whole Fruit	−0.109	−0.101	−0.094	−0.105	−0.079	−0.076	−0.013	0.065	−0.031	−0.045	−0.028	−0.052	0.109	−0.030
Total Vegetables	−0.024	0.025	0.032	0.024	0.089	0.074	0.020	−0.025	0.013	0.047	0.034	0.119	−0.007	−0.030
Greens and Beans	0.057	0.097	0.070	0.089	0.103	0.050	−0.003	−0.049	0.040	0.107	0.065	0.134	0.057	0.059
Whole Grains	0.038	0.040	0.044	0.027	−0.067	−0.001	−0.026	−0.046	0.001	0.066	0.047	0.008	0.141 *	−0.045
Dairy	0.014	0.014	0.013	0.016	0.023	0.055	0.008	0.110	0.004	−0.066	0.011	−0.065	0.068	−0.020
Total Protein Foods	0.000	0.019	−0.023	0.005	−0.012	−0.010	−0.054	−0.025	0.014	−0.052	−0.062	−0.133	−0.029	0.049
Seafood and Plant Proteins	0.028	0.006	0.033	0.032	−0.012	−0.056	−0.014	0.027	−0.015	−0.007	−0.021	0.028	0.013	0.027
Fatty Acids	0.075	0.067	0.057	0.074	0.140 *	0.053	−0.043	0.024	−0.073	−0.068	−0.065	−0.002	−0.069	−0.089
Moderation Components														
Refined Grains	−0.050	−0.041	0.026	−0.040	−0.020	−0.033	−0.038	−0.013	−0.054	−0.010	−0.010	0.123	0.018	0.048
Sodium	−0.026	−0.022	0.009	−0.012	−0.112 *	−0.051	0.078	−0.038	0.099	0.090	0.120	0.062	0.085	0.082
Empty Calories (HEI-2010)	−0.035	−0.017	−0.035	−0.006	−0.025	−0.052	0.035	0.005	0.059	0.013	0.026	−0.094	0.093	−0.119 *
Added Sugars (HEI-2015)	0.032	0.015	0.040	0.002	0.030	0.055	−0.043	0.022	−0.068	−0.038	−0.054	0.077	−0.069	0.101
Saturated Fats (HEI-2015)	−0.053	−0.061	−0.103	−0.027	−0.009	−0.025	0.028	−0.048	0.060	0.098	0.097	0.051	−0.123 *	0.070
Total HEI Score														
HEI-2010	−0.027	0.001	0.025	0.007	−0.031	−0.034	0.026	0.000	0.048	0.060	0.085	0.052	0.179 **	−0.040
HEI-2015	−0.015	0.002	0.034	0.005	−0.022	−0.006	−0.016	−0.002	0.010	0.053	0.065	0.127	0.106	0.088

* Correlation is significant at the 0.05 level (two-tailed). ** Correlation is significant at the 0.01 level (two-tailed). BMI, body mass index; BF%, percentage body fat; WC, waist circumference; SBP, systolic blood pressure; DBP, diastolic blood pressure; TC, total cholesterol; LDL, low-density lipoprotein; HDL, high-density lipoprotein; TG, triglyceride; MVPA, moderate-to-vigorous physical activity; ST, screen time.

**Table 6:** Association between poor HEI-2010 and HEI-2015 total scores and CVD risk factors in schoolchildren.

**HEI-2010 Poor Total Score ≤ 50 Points**												
							**Adjustment for Sex, MVPA, and ST**					
**Risk Factor**	**B**	**SE**	**Sig.**	**Exp(B)**	**95% CI for Exp(B)**		**B**	**SE**	**Sig.**	**Exp(B)**	**95% CI for Exp(B)**	
					**Lower**	**Upper**					**Lower**	**Upper**
Obesity +2 SD	−1.057	0.202	0.000	0.347	0.234	0.516	−0.310	0.258	0.229	0.733	0.442	1.216
Elevated BP	−0.595	0.311	0.056	0.552	0.300	1.016	−0.174	0.327	0.594	0.840	0.443	1.594
Stage 1 HTN	0.511	0.730	0.484	1.667	0.398	6.974	1.001	0.756	0.185	2.722	0.619	11.976
Stage 2 HTN	−21.203	28,420.722	0.999	0.000	0.000	0.000	−0.174	0.327	0.594	0.840	0.443	1.594
**HEI-2015 Poor Total Score ≤ 50 Points**												
							**Adjustment for Sex, MVPA, and ST**					
**Risk Factor**	**B**	**SE**	**Sig.**	**Exp(B)**	**95% CI for Exp(B)**		**B**	**SE**	**Sig.**	**Exp(B)**	**95% CI for Exp(B)**	
					**Lower**	**Upper**					**Lower**	**Upper**
Obesity +2 SD	−0.578	0.184	0.002	0.561	0.391	0.805	−0.105	0.241	0.665	0.901	0.561	1.446
Elevated BP	0.442	0.427	0.301	1.556	0.673	3.594	0.804	0.446	0.071	2.235	0.932	5.365
Confounder												
Sex							−0.744	0.200	0.000	0.475	0.321	0.703
Stage 1 HTN	0.000	0.707	1.000	1.000	0.250	3.998	0.300	0.722	0.677	1.350	0.328	5.556
Stage 2 HTN	0.000	1.414	1.000	1.000	0.063	15.988	0.358	1.440	0.804	1.430	0.085	24.069

Binary logistic regression with boys as the reference group. Variables entered in step 1: sex, meeting MVPA; moderate-to-vigorous physical activity; and meeting ST, screen time.

## Data Availability

The data presented in this study are available on request from the corresponding author. The data are not publicly available due to privacy reasons.
